# Oral Nitrate and Platelet Reactivity: Do Men and Women Respond Differently?∗

**DOI:** 10.1016/j.jacbts.2021.11.006

**Published:** 2022-01-24

**Authors:** Albert Ferro

**Affiliations:** School of Cardiovascular Medicine and Sciences, British Heart Foundation Centre of Research Excellence, King’s College London, London, United Kingdom

**Keywords:** nitrate, nitric oxide, nitrite, platelet, sex

**Figure:**
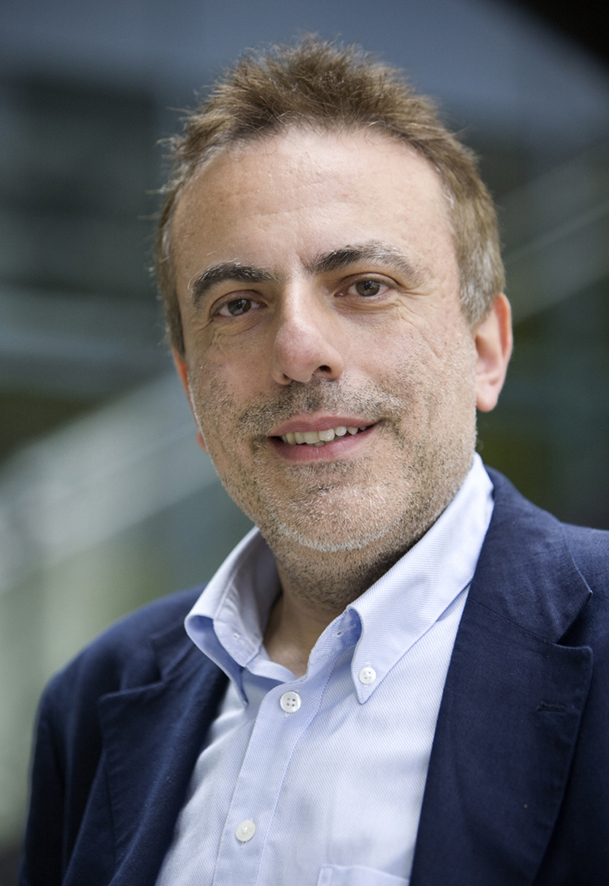

We stand at a pivotal moment in world history, where the dangers of climate change and resultant global warming are very much in the news and measures to address this are urgently being discussed by world leaders. It is widely accepted that livestock are a major emitter of greenhouse gases, and the benefits of moving to plant-based diets are being extolled for the future health of our planet. Aside from the global implications, plant-based diets may promote better health at the individual level, being associated with less cancers, digestive diseases, type 2 diabetes, and obesity, as well as less cardiovascular disease (1).

The paper by Godwin et al (2) in this issue of *JACC: Basic to Translational Science* is a noteworthy contribution to the cardiovascular narrative, specifically examining the effect of oral nitrate (with which green leafy vegetables are enriched) on platelet function in healthy young people, and additionally looking at whether these effects differ between the sexes. The authors report a number of findings. First, platelets from women are more reactive than those from men in the basal state (that is to say, after treatment with placebo). Second, an oral nitrate load decreases platelet reactivity in women, but in men it does not decrease platelet reactivity and, according to which assay is used, may actually augment it. Third, the oral nitrate load increases both plasma nitrite plus nitrate and platelet nitric oxide (NO) production, and the latter of these increases is considerably greater in magnitude in women than in men (10-fold vs 2-fold). Interestingly, in women, enteric processing and absorption of nitrate appears to be necessary for it to augment platelet NO production, possibly through interaction with the gut microbiome (although this was not specifically investigated), whereas this is not the case in men. These findings highlight apparent sex differences in oral nitrate handling and subsequent effects on platelet NO generation, as well as in effects of oral nitrate on overall platelet reactivity.

At first sight, the divergent effects of nitrate on NO production and activation state of platelets appear somewhat puzzling. NO is generally believed to inhibit platelet activity and to be antithrombotic. However, evidence also suggests that NO at lower concentrations may paradoxically activate platelets, whereas at higher concentrations it does the opposite, and that both the activatory and inhibitory effects are cGMP-mediated (3). The relationship between ambient NO concentration in response to nitrate ingestion and platelet function may therefore be quite a complex one, not just between the sexes, but even within a given individual depending on their dietary nitrate content at any given time. It is also possible that nitrate ingestion is giving rise to the generation not only of NO but of other chemical entities that can counteract the antiactivatory effects of NO, such as peroxynitrite (ONOO^−^), formed by the reaction of NO with superoxide anion (O_2_^−^). Indeed, there is evidence that, at least premenopausally, women generate less reactive oxygen species than do men, possibly related to antioxidant properties of estrogen, sex differences in NADPH-oxidase activity, or other mechanism(s) yet to be defined (4).

The physiological relevance of platelet NO generation continues to be a subject of controversy. Although platelets undoubtedly contain the endothelial-type NO synthase enzyme, it is not clear whether this is vestigial or contributes to ambient NO concentrations in the circulation to a physiologically relevant extent. What seems beyond question is that the vast majority of NO in the circulation is released from the vessel wall rather than from platelets. In the present study, the sex difference in the effect of oral nitrate on platelet NO production was much greater in percentage or fold terms than that on plasma nitrite/nitrate, again underscoring the fact that platelet-derived NO only makes a marginal contribution to circulating NO. However, locally produced NO by platelets may have important autocrine, or indeed (within the developing thrombus) paracrine, effects on thrombus progression (5). The physiological and pathophysiological importance of the effects on platelet NO generation described in this paper is therefore difficult to disentangle.

It also must be borne in mind that nitrate is only one of a plethora of substances present within plants that can confer important benefits to the cardiovascular system, and that it is present in important amounts in only certain types of plants, namely the green leafy kind. Polyphenols of various kinds, including flavonoids, phenolic acids, and tannins, are the most abundant antioxidants in plants, and may at least in theory play significant roles in cardiovascular protection through removal of damaging reactive oxygen species, although this remains to be proven. Sulforaphane is an organosulfur compound found in cruciferous vegetables (such as broccoli, brussels sprouts, cabbage, cauliflower, and kale), which activates antioxidant and anti-inflammatory pathways through Nrf2 induction and NF-κB inhibition; again, although these actions may theoretically exert cardiovascular and other health benefits, these remain unproven. Similar considerations apply to antioxidant vitamins and alkaloids present within a wide variety of plants.

The present paper was performed in a relatively small (n = 22) cohort of young healthy individuals. To confirm its importance in physiology and pathophysiology, further work in this line will need to address both older healthy individuals and those with cardiovascular risk factors—and, indeed, patients with established atherosclerotic disease. Nonetheless, it presents an interesting perspective on the role of dietary nitrate in modulating platelet function and thereby atherosclerosis and its thrombotic complications. It also highlights that its role is not likely to be straightforward. The effects demonstrated here are also likely to be only one of a multitude of ways in which plant-based foods impinge on cardiovascular function in both health and disease. Whether oral nitrate supplementation may have therapeutic value in treating or preventing atherothrombotic disease remains to be determined. If it does, the data presented here suggests that it will need to be tailored to the individual patient, and in particular, that there may be a sex difference in any potential benefit of such a therapeutic strategy.

## Funding Support and Author Disclosures

The author has reported that he has no relationships relevant to the contents of this paper to disclose.
